# Cytoprotective Potential of Fucoxanthin in Oxidative Stress-Induced Age-Related Macular Degeneration and Retinal Pigment Epithelial Cell Senescence In Vivo and In Vitro

**DOI:** 10.3390/md19020114

**Published:** 2021-02-18

**Authors:** Shiu-Jau Chen, Tzer-Bin Lin, Hsien-Yu Peng, Hsiang-Jui Liu, An-Sheng Lee, Cheng-Hsien Lin, Kuang-Wen Tseng

**Affiliations:** 1 Department of Neurosurgery, Mackay Memorial Hospital, Taipei 10449, Taiwan; T149@mmc.edu.tw; 2 Department of Medicine, Mackay Medical College, New Taipei 25245, Taiwan; hypeng@mmc.edu.tw (H.-Y.P.); anshenglee@mmc.edu.tw (A.-S.L.); davidlin@mmc.edu.tw (C.-H.L.); 3 Department of Physiology, School of Medicine, College of Medicine, Taipei Medical University, Taipei 11049, Taiwan; tblin2@tmu.edu.tw; 4 Department of Optometry, Mackay Junior College of Medicine, Nursing, and Management, New Taipei 11260, Taiwan; s458@eip.mkc.edu.tw

**Keywords:** fucoxanthin, retinal pigment epithelium, premature senescence, age-related macular degeneration

## Abstract

Oxidative stress is identified as a major inducer of retinal pigment epithelium (RPE) cell dysregulation and is associated with age-related macular degeneration (AMD). The protection of RPE disorders plays an essential role in the pathological progress of retinal degeneration diseases. The pharmacological functions of fucoxanthin, a characteristic carotenoid, including anti-inflammatory and antioxidant properties, may ameliorate an outstanding bioactivity against premature senescence and cellular dysfunction. This study demonstrates that fucoxanthin protects RPE cells from oxidative stress-induced premature senescence and decreased photoreceptor cell loss in a sodium iodate-induced AMD animal model. Similarly, oxidative stress induced by hydrogen peroxide, nuclear phosphorylated histone (γH2AX) deposition and premature senescence-associated β-galactosidase staining were inhibited by fucoxanthin pretreatment in a human RPE cell line, ARPE-19 cells. Results reveal that fucoxanthin treatment significantly inhibited reactive oxygen species (ROS) generation, reduced malondialdehyde (MDA) concentrations and increased the mitochondrial metabolic rate in oxidative stress-induced RPE cell damage. Moreover, atrophy of apical microvilli was inhibited in cells treated with fucoxanthin after oxidative stress. During aging, the RPE undergoes well-characterized pathological changes, including amyloid beta (Aβ) deposition, beta-site amyloid precursor protein-cleaving enzyme 1 (BACE1) expression and tight junction disruption, which were also reduced in fucoxanthin-treated groups by immunofluorescence. Altogether, pretreatment with fucoxanthin may protect against premature senescence and cellular dysfunction in retinal cells by oxidative stress in experimental AMD animal and human RPE cell models.

## 1. Introduction

While aging causes a decline in the ability to respond and adapt to the accumulative impact of different exposures, age-related disease develops when cellular dysfunction from compromised cytoprotective pathways is severe enough to cause tissue destruction [1]. Aging is also an independent risk for visual impairment. With aging, the photoprotective capability of the retinal pigment epithelium (RPE) decreases. During aging, the RPE undergoes well-characterized structural changes, including apical microvilli atrophy, accumulation of formation of drusen and progressive cell loss [2]. Many cellular stresses activate senescence, a terminal arrest of proliferation, including dysfunctional telomeres, DNA damage and oxidative stress.

Age-related macular degeneration (AMD) is a leading cause of blindness affected by oxidative stress among the elderly, characterized by RPE degeneration. Histological changes in RPE cells, including atrophy of apical microvilli and disruption of cellular junctions, occur before the loss of photoreceptor cells [3]. Sodium iodate-induced retinal degeneration animal models, which selectively destroy the RPE and elicit AMD-associated characteristics, have been widely used to investigate the mechanisms involved in retina degeneration for human retinal disorders because of the similar RPE and photoreceptor degeneration to that noticed in patients with AMD [4,5,6]. The RPE and photoreceptors of the retinal tissue comprise organized symbiotic and interconnected metabolic relationships. The RPE, sited between the outer segments of the photoreceptors and blood supply of the choroid, continuously removes waste and provides nutrients for neighboring photoreceptors. The RPE operates specialized roles critical for homeostasis and metabolism of the neural retina, including immune modulation and the visual cycle. Another specialized function of the RPE is to provide nourishment and phagocytosis of photoreceptors which are in intimate contact with the microvilli [7]. One RPE cell affords assistance for about 40 adjacent photoreceptors [8]. The tight junction is the foundation stone of the RPE barrier. Tight junctions form a belt that completely encircles cells of the epithelial monolayer. Historically, epithelia have been classified as leaky or tight in accordance with ions, which cross the epithelium via the paracellular space relative to transport across the cells themselves. RPE tight junctions are interrupted in a variety of ocular disorders such as macular degeneration [9,10].

The RPE is derived from the outer layer of the optic cup that grows out from the forebrain. Therefore, AMD, a devastating neurodegenerative disease, has many pathological characteristics that are common to Alzheimer’s disease, including amyloid beta (Aβ) accumulation, which has also been demonstrated to be associated with drusen in eyes [11,12]. Elevated Aβ levels have been observed in elderly retinal tissues, and Aβ has been shown to be associated with the development of AMD [13]. Aβ1-42, a component of drusen, contributes to inflammation, disturbed RPE morphology and function in AMD [14]. The strongest known risk factor for AMD is advanced age, with the risk of developing AMD with increasing senescence [15]. Other identified risk factors for AMD include oxidative stress, smoking, hypertension, hypercholesterolemia and diet [16]. It has been demonstrated that hydrogen peroxide and low-density lipoprotein (LDL) are associated with the development of AMD in vivo and in vitro [17,18,19]. Reactive oxygen species (ROS) in turn damage mitochondria and lipids, leading to alteration of retinal function and visual impairment.

Generally, oxidative stress is characterized by increased levels of ROS, resulting in its damage. ROS, such as hydrogen peroxide, that extensively attack DNA, cellular junctions and other cellular organelles play an important physiological and pathophysiological role in controlling various cellular functions such as cellular differentiation, proliferation, senescence and death. Further, ROS are important signaling molecules that play an essential role in the progression of inflammation. Malondialdehyde (MDA) expression and nuclear phosphorylated histone (γH2AX) deposition were used as markers of lipid peroxidation and DNA strand damage. Administration of antioxidants targeting ROS generation and reducing ROS-induced cell damage of the RPE may prevent AMD progression [20,21]. Catalase, superoxide dismutase and glutathione peroxidase protect RPE cells through increased expression by effectively scavenging ROS and attenuating oxidative damage [22,23]. These enzymes can quickly scavenge ROS and protect the body from injuries caused by active substances or toxic substances.

**Figure 1:**
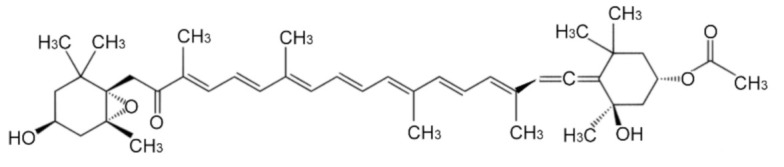
The chemical structure of fucoxanthin.

Fucoxanthin (Figure 1) is an orange-colored pigment present in brown seaweeds, such as *Hijikia fusiformis*, *Laminaria japonica* and *Sargassum fulvellum* [24], and responsible for the higher antioxidant activity. The biological activities of fucoxanthin have been reported to have antioxidant, anti-inflammatory, anticancer and antimicrobial effects in various tissues and cells, as tested by in vitro or in vivo experiments [25,26,27,28]. We demonstrated that pretreatment with fucoxanthin inhibited the ultraviolet B-induced corneal inflammatory pain and thinning of the corneal epithelial layer [29,30]. Due to the necessary functions that keep photoreceptors healthy, the RPE is critical for maintaining eye vision; however, the effects of fucoxanthin have not been extensively examined on oxidative stress-induced AMD and premature senescence. Therefore, we conducted this in vivo and in vitro study to examine the effect of fucoxanthin on the premature senescence of RPE cells challenged by hydrogen peroxide and investigated the possible mechanisms underlying this effect.

## 2. Results

### 2.1. Fucoxanthin Pretrement Inhibits ROS Generation and Lipid Peroxidation in Sodium Iodate-Induced Retinal Degeneration Animal Model

**Figure 2:**
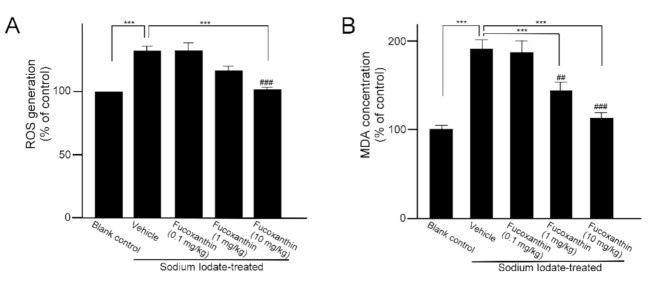
Effects of fucoxanthin on ROS (**A**) generation and MDA concentration (**B**) in sodium iodate-induced retinal degeneration. The results are represented as the mean ± SD (*n* = 5). The mean value was significantly different as compared with the sodium iodate/vehicle group (*** *p* < 0.01; Student’s *t* test). The mean value was significantly different in the sodium iodate-induced group (## *p* < 0.05 and ### *p* < 0.01; one-way ANOVA followed by Bonferroni’s multiple comparison test). ROS, reactive oxygen species; MDA, malondialdehyde.

The main representative of the retinal disorder induced by sodium iodate is significant and progressive damage to the RPE. Otherwise, a significant decrease in photoreceptors was also observed in the outer nuclear layer. To investigate the protective effect of fucoxanthin against sodium iodate-induced retinal degeneration, experimental animals were treated daily with fucoxanthin over a period of 1 week before sodium iodate-induced retinal degeneration. ROS generation and the MDA concentration of the retinal tissues are indicative of the oxidative damage capacity, respectively. ROS levels in retinas of the sodium iodate-treated rats were markedly increased as compared with the blank control group. However, the group pretreated with 10 mg/kg BW fucoxanthin indicated a significant decrease in ROS generation compared with the sodium iodate/vehicle group (Figure 2A). In addition, the concentrations of MDA in retinal tissues were analyzed. The MDA levels in the ocular tissue of the sodium iodate/vehicle group were significantly increased as compared with the blank control group, but the levels were significantly decreased in groups pretreated with 1 (*p* < 0.05) and 10 mg/kg fucoxanthin (*p* < 0.01) (Figure 2B).

### 2.2. Fucoxanthin Inhibits Cellular Senescence in Retinal Tissues of Sodium Iodate-Induced Retinal Degeneration In Vivo

**Figure 3:**
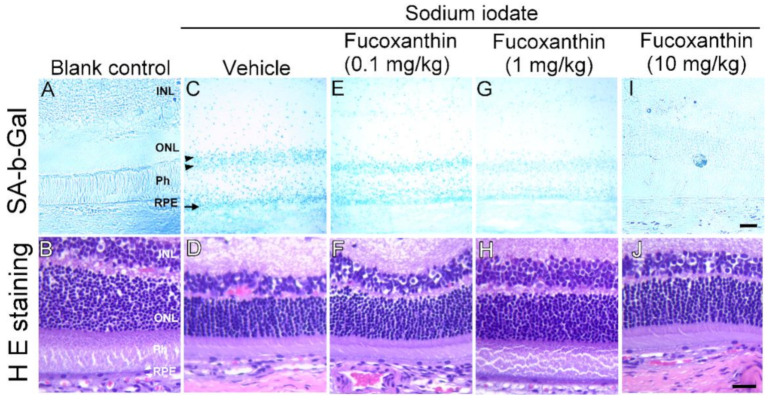
Inhibited prosenescent effect of fucoxanthin on retinal cells of sodium iodate-induced retinal degeneration animal model. SA-b-Gal staining and histological changes were compared with blank control (**A**,**B**), sodium iodate/vehicle (**C**,**D**), sodium iodate/fucoxanthin at 0.1 mg/kg (**E**,**F**), sodium iodate/fucoxanthin at 1 mg/kg (**G**,**H**) and sodium iodate/fucoxanthin at 10 mg/kg (**I**,**J**) groups. The intense blue deposits of SA-b-Gal staining (**C**) and remarkably thin outer nuclear layer (ONL) (**D**) were observed in retinas of sodium iodate-induced experimental animals, as compared to those of the blank control group (**A**,**B**). In contrast, there were limited stained cells (**I**) and increased thickness of the ONL (**J**) with fucoxanthin treatment at 1 mg/kg as compared with retinal cells of the sodium iodate/vehicle group. H E staining, hematoxylin and eosin staining; INL, inner nuclear layer; ONL, outer nuclear layer; Ph, photoreceptor layer; RPE, retinal pigment epithelium; SA-b-Gal, senescence-associated β-galactosidase. Scale bars: 40 μm.

To examine inhibitory prosenescent properties of fucoxanthin in vivo, we pretreated the animals with fucoxanthin (0.1, 1 and 10 mg/kg/day) for seven days before sodium iodate-induced retinal degeneration and examined retinal senescence and histological changes. β-Galactosidase (β-gal), a lysosomal hydrolytic enzyme with the physiological function of catalyzing the hydrolysis of glycosidic bonds which transform lactose into galactose, is known to be characteristic of senescent cells. The intense blue deposits of senescence-associated β-galactosidase (SA b-Gal) staining were observed in sodium iodate-induced experimental animals (Figure 3C), comparable with that of the blank control group (Figure 3A). Further, a marked decrease in photoreceptors and thinning in the outer nuclear were found in retinal degeneration animals as compared with the blank control group (Figure 3D). These findings agree with our SA-b-Gal staining results. Although sodium iodate treatment caused severe retinal generation, sodium iodate effects were inhibited by fucoxanthin. Significant improvements in the prosenescent properties and histological changes were observed during examination of the group treated with 10 mg/kg fucoxanthin compared (Figure 3I,J) with the retinal disorders detected in the sodium iodate-treated experimental animals (Figure 3A,B).

### 2.3. Fucoxanthin Affects Oxidative Stress-Induced ROS Generation and Mitochondria Respiration

**Figure 4:**
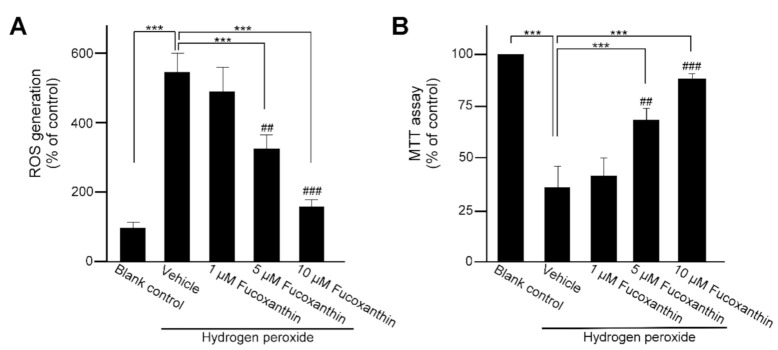
Effects of fucoxanthin on cultured human ARPE-19 cells’ viability. After treatment with fucoxanthin (1, 5 and 10 μM) for 24 h, ARPE-19 cells were treated with hydrogen peroxide exposure. Cell viability was analyzed by ROS generation (**A**) and MTT (**B**) assay. The value of the control group was considered 100%. Values presented are mean ± SD (*n* = 5). The mean value was significantly different as compared with the hydrogen peroxide/vehicle group (*** *p* < 0.01; Student’s *t* test). The mean value was significantly different in the hydrogen peroxide exposure group (## *p* < 0.05 and ### *p* < 0.01; one-way ANOVA followed by Bonferroni’s multiple comparison test). ROS, reactive oxygen species; MTT, 3-(4,5-dimethylthiazol-2-yl)-2,5-diphenyltetrazolium bromide.

To investigate the cytoprotective effect of fucoxanthin and hydrogen peroxide in human ARPE-19 cells, the cells were pretreated with 1, 5 or 10 μM fucoxanthin for 48 h and with 500 μM hydrogen peroxide for an additional 48 h. Hydrogen peroxide increased the ROS level in the ARPE-19 cells and fucoxanthin exhibited an inhibitory effect on oxidative stress-induced ROS production. Compared with the ROS generation detected in the hydrogen peroxide-exposed groups, drastically decreased ROS generation was observed in the group treated with 5 and 10 μM fucoxanthin (Figure 4A). Moreover, mitochondria respiration was determined by the 3-(4,5-dimethylthiazol-2-yl)-2,5-diphenyltetrazolium bromide (MTT) assay. Compared with hydrogen peroxide-exposed groups, mitochondria respiration was increased in fucoxanthin pretreatment groups and a marked difference was observed in 5 and 10 μM fucoxanthin groups (Figure 4B). Thus, fucoxanthin is shown to suppress ROS generation and promote mitochondria respiration.

### 2.4. Fucoxanthin Protects ARPE-19 Cells from Hydrogen Peroxide-Induced Cellular Senescence and DNA Damage Response

**Figure 5:**
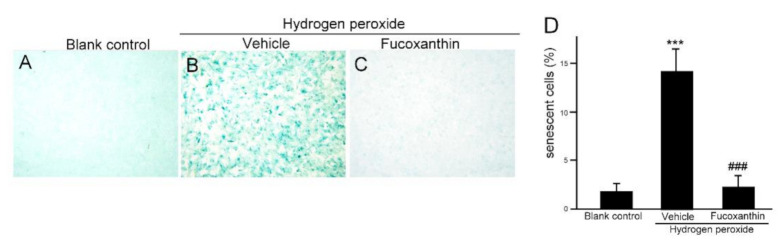
Effects of fucoxanthin on prosenescent properties after hydrogen peroxide exposure in vitro. SA-b-Gal staining of ARPE-19 was compared between the following groups: blank control (**A**), hydrogen peroxide (**B**) and hydrogen peroxide/10 μM fucoxanthin (**C**). Positively stained cells were quantified and are shown as percentages of the total number of cells and the results are presented as means ± SD (**D**). (*** *p* < 0.01 as compared with blank control group; ### *p* < 0.01 as compared with hydrogen peroxide exposure group; Student’s *t* test). SA-b-Gal, senescence-associated β-galactosidase.

Hydrogen peroxide is a potent senescence inducer and plays an important role in the induction of senescence. It is well established that hydrogen peroxide triggers potent senescence and DNA damage by various signaling cascades through oxidative stress. To investigate whether fucoxanthin protects against oxidative stress-induced cellular senescence and DNA damage, ARPE-19 cells were pretreated with fucoxanthin for 24 h and then exposed to 500 μM hydrogen peroxide for another 24 h. The cellular senescence was determined with galactosidase activity. The proportion of intense blue deposits after SA-b-Gal staining cells exhibited a statistically significant increase in the ARPE-19 cultures treated with 500 μM hydrogen peroxide for 24 h alone (Figure 5B), compared with the untreated control cultures (Figure 5A). However, the ARPE-19 cells pretreated with 10 μM fucoxanthin for 24 h before hydrogen peroxide exposure exhibited a reduced number of hydrogen peroxide-induced SA-b-Gal-stained cells compared with the hydrogen peroxide group (Figure 5C,D). This result suggests that fucoxanthin could inhibit hydrogen peroxide-induced cellular senescence in ARPE-19 cells.

**Figure 6:**
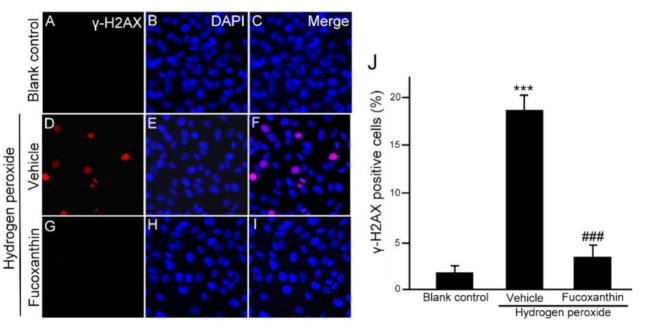
Effects of fucoxanthin on DNA damage after hydrogen peroxide exposure. Immunofluorescence staining of nuclear γH2AX was compared between the following groups: blank control (**A**–**C**), hydrogen peroxide (**D**–**F**) and hydrogen peroxide/fucoxanthin (**G**–**I**). DAPI counterstaining shows the nuclear localization of γH2AX. Positively stained cells were quantified and are shown as percentages of the total number of cells and the results are presented as means ± SD (**J**). (*** *p* < 0.01 as compared with blank control group; ### *p* < 0.01 as compared with hydrogen peroxide exposure group; Student’s *t* test). γH2AX, nuclear phosphorylated histone. DAPI, 40,6-diamidino-2-phenylindole.

Moreover, there are well-known molecular triggers for the senescence response, including the DNA damage response, so we also examined the protective effect of fucoxanthin from DNA damage in ARPE-19 cells after oxidative stress. Immunofluorescence staining for γH2AX, the substitute marker of the DNA damage reaction, revealed that oxidative stress commanded to nuclear cH2AX deposition, compared with the significant increase in nuclear γH2AX observed in the hydrogen peroxide-treated groups (Figure 6D–F), which was reduced with fucoxanthin treatment (Figure 6G–I). These results demonstrate that fucoxanthin suppresses ARPE-19 cells from oxidative stress-induced DNA damage (Figure 6J). 

### 2.5. Fucoxanthin Promotes Cell Junction and Morphogenesis of Apical Microvilli

**Figure 7:**
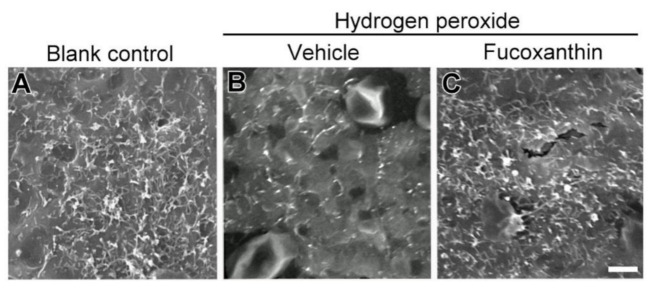
Morphological changes of apical microvilli by scanning electron microscopy. Ultrastructure of microvilli in a monolayer of cultured ARPE-19 cells was compared between the following groups: blank control (**A**), hydrogen peroxide (**B**) and hydrogen peroxide/fucoxanthin (**C**). ARPE-19 cells displayed an apical surface densely covered by microvilli (**A**). After hydrogen peroxide exposure, the number of apical microvilli was decreased as compared with the blank control group. In contrast, not only the number, but also the morphology of microvilli was affected by the treatment of the cultured cells with fucoxanthin. Scale bars: 5 μm.

To test the cell structure protective effects of fucoxanthin, scanning electron microscopy (SEM) was used to investigate the ultrastructure morphological changes. The intact cell junction of cultured cells was observed by electron microscopy. The RPE operates specialized metabolic and transport functions critical for homeostasis, thereby forming a part of the blood–retina barrier. The apical surface of RPE cells radiates long and thin microvilli that establish a complex of close structures (Figure 7A). The cellular morphological changes reflected that RPE senescence occurred. The effects of losing RPE–RPE adhesion and the broad space between adjoining cells after hydrogen peroxide exposure were also investigated. Some cells shrank and became round. The elaboration of apical microvilli presented a decrease in hydrogen peroxide-treated RPE cells as compared with blank control cells. Stubby apical microvilli (Figure 7B) were revealed. Compared with the hydrogen peroxide-exposed group, mildly disordered cell junctions and remarkably long microvilli were demonstrated in the fucoxanthin-pretreated group (Figure 7C). These results suggest that fucoxanthin may protect the RPE cells from essentially changed microvilli and cell junctions induced by oxidative stress.

### 2.6. Fucoxanthin Protects Hydrogen Peroxide-Induced Degradation of Cytoskeleton Actin C and Disrupyion of Cell Junction

**Figure 8:**
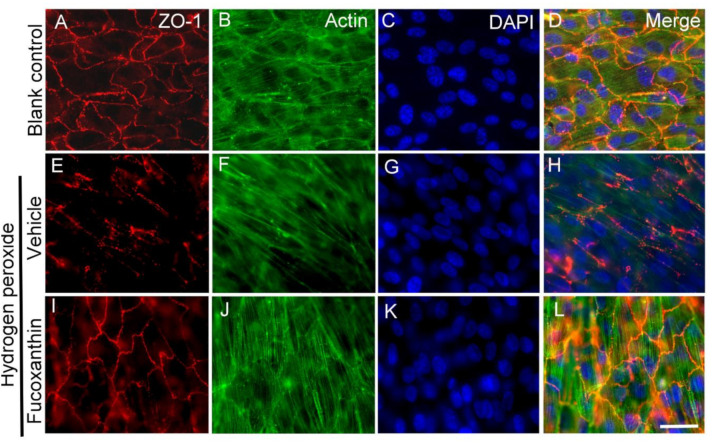
Effects of fucoxanthin on cell junction and cytoskeleton structure after exposure to hydrogen peroxide. ARPE-19 cells were stained with antibodies against ZO-1 and actin, and nuclei were counterstained with DAPI in blank control (**A**–**D**), hydrogen peroxide/vehicle (**E**–**H**) and hydrogen peroxide/fucoxanthin groups (**I**–**L**). Immunostaining showed a con-tinuous labeling of ZO-1 in the region of cell–cell contacts and a filamentous structure of actin in the cytoplasm in cells of the blank control group. Marked disruption of ZO-1 (**E**) and disorganized actin (**F**) were observed after hydrogen peroxide exposure. In contrast, the cells showed a more continuous cell junction (**I**) and filamentous cytoskeleton (**K**) in the cells with fucoxanthin treatment. DAPI, 40,6-diamidino-2-phenylindole. Scale bar: 10 μm.

As the abovementioned experiment observed the protective effect of fucoxanthin on microvilli formation following exposure to hydrogen peroxide, further study was performed to examine the morphogenesis of the cytoskeleton and cell junction. The effect of fucoxanthin on the fluorescence staining of tight junction protein zonular occludens (ZO-1) and F-actin in ARPE-19 exposure to hydrogen peroxide was investigated by immunofluorescence microscopy. The results show that the expression continuous around the ZO-1 cells and the filamentous structure of actin were observed in the ARPE-19 monolayer culture (Figure 8A–D). With hydrogen peroxide exposure, cell junction networks and filamentous actin collapsed rapidly in the ARPE-19 cells. The abnormal distribution of ZO-1 typically manifested as fragmental staining and actin was charlatanically exhibited as diffused staining (Figure 8E–H). An organized cell junction and filamentous cytoskeleton were detected in fucoxanthin-pretreated ARPE-19 cells (Figure 8I–L). From this examination, we found that significantly protective effects on the cell junction and actin cytoskeleton structure can be observed from cells pretreated with fucoxanthin.

### 2.7. Fucoxanthin Inhibits Hydrogen Peroxide Exposure Up-Regulated Cellular Expressions of Aβ1-42 and Beta-Site Amyloid Precursor Protein-Cleaving Enzyme 1 (BACE1)

**Figure 9:**
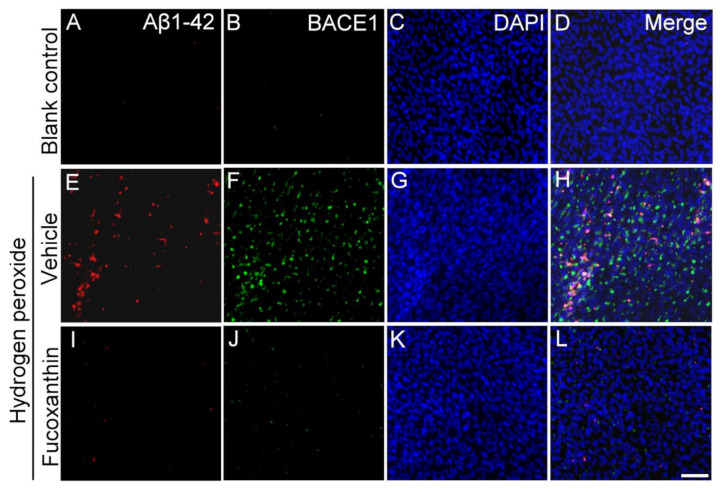
Effects of fucoxanthin on the expression of AMD-related proteins Aβ1-42 and BACE1 after exposure to hydrogen peroxide. Immunofluorescence staining of Aβ1-42 and BACE1 was compared between the following groups: blank control (**A**–**D**), hydrogen peroxide (**E**–**H**) and hydrogen peroxide/fucoxanthin (**I**–**L**). Expression of AMD-related proteins Aβ1-42 (**A**) and BACE1 (**B**) is limited in cells of the blank control group. Immunohistochemical staining showed evident induction of Aβ1-42 (**E**) and BACE1 (**F**) expression after exposure to hydrogen peroxide. In contrast, the experimental animals showed decreased expressions of Aβ1-42 (**I**) and BACE1 (**J**) with fucoxanthin treatment. Scale bar: 40 μm.

BACE1 is a transmembrane protease responsible for the β-site cleavage of the amyloid precursor protein to produce Aβ, and Aβ is an important component of plaques in neurological disease and drusen deposits in AMD. To test the protective effect of fucoxanthin on the AMD cellular model, fucoxanthin was added to ARPE-19 cells exposed to hydrogen peroxide. To further confirm the involvement of AMD, the expression of its various drusen-related proteins including Aβ1-42 and BACE1 was examined by immunofluorescence assays. Hydrogen peroxide exposure up-regulated cellular expressions of Aβ1-42 and BACE1 (Figure 9E–H) compared with the control group (Figure 9A–D). These results reveal that hydrogen peroxide activated the expression of drusen-related proteins in ARPE-19 cells. However, the increased expressions of Aβ1-42 and BACE1 were consequently down-regulated with fucoxanthin pretreatment (Figure 9I–L), suggesting a protective role of fucoxanthin on the ARPE-19 cells in reducing formation of Aβ deposition.

## 3. Discussion

This study demonstrated that fucoxanthin has a cytoprotective effect on retinal cell degeneration in a dose–response fashion in experimental animal and cultured cell models. Treatment with fucoxanthin substantially inhibited the DNA strand damage marker, and nuclear γH2AX deposition and premature SA β-galactosidase staining were observed following fucoxanthin pretreatment. An administration of fucoxanthin significantly inhibited ROS generation, reduced MDA concentrations and increased the mitochondrial metabolic rate in oxidative stress-induced RPE cell damage. Moreover, atrophy of apical microvilli, Aβ deposition, Beta-secretase 1 (BACE1) expression and tight junction disruption were also reduced and inhibited in cells treated with fucoxanthin after oxidative stress.

AMD is a complex eye disease and is classified into wet or dry forms. Wet AMD is characterized by the sprouting of new vessels from choriocapillaris through Bruch’s membrane. Drusen and RPE alterations are the hallmark of dry AMD [31]. Oxidative stress plays an important role in the development of AMD. As revealed, RPE degeneration, accumulation of lipofuscin, formation of drusen and microvilli atrophy have been associated with the pathogenesis of dry AMD in relation to increased oxidative stress [32]. Oxidative stress is also a potent inducer of inflammatory cytokines promoting inflammation and macrophage infiltration in retinal tissues [33]. Studies revealed significant changes in fucoxanthin-regulated oxidative stress and inflammatory responses in various tissues [29,34]. To elucidate the potential protective effects against oxidative stress in retinal cells, ROS generation and the peroxidation index were measured. In this work, fucoxanthin provided protection against hydrogen peroxide-induced ROS in human RPE cells. Sodium iodate- and hydrogen peroxide-induced ROS are well known for studying RPE cell senescence models [15,35,36]. Here, our results demonstrate that hydrogen peroxide can lead to cell senescence and increased ROS generation, MDA production and DNA damage in human RPE cells, but this process was substantially inhibited with fucoxanthin treatment. These findings suggest that fucoxanthin reduces hydrogen peroxide-triggered cell senescence in human RPE cells, which may be strongly correlated with the antioxidative effects of fucoxanthin.

Mitochondrial dysfunction and oxidative damage are appreciably improved with senescence and aging-related diseases. During aging, increased ROS disrupt mitochondrial DND, lipids and structure and limit energy production in AMD [37,38,39]. ROS and imbalanced mitochondrial calcium cause the mitochondrial permeability transition pore to open and lead to cell death [40]. Studies demonstrated that mitochondrial function is significantly affected by AMD in RPE cells and mitochondrial disorders are involved in AMD pathology [41,42]. Oxidative stress-induced mitochondrial damage of the RPE has a secondary effect on photoreceptors in the AMD progression [43]. Here, mitochondria respiration was determined by the MTT assay. Compared with hydrogen peroxide-exposed groups, mitochondria respiration was increased in human RPE cells of the fucoxanthin pretreatment groups Thus, fucoxanthin is shown to inhibit ROS generation and promote mitochondria function.

The actin cytoskeleton is a highly dynamic structure that participates in the morphogenesis of apical microvilli. Due to phototoxicity, the daily renewal of the outer segment of the photoreceptor is ensheathed by microvilli arising from the surface of the pigment epithelial cells and is intensely important to the survival of photoreceptors [7,44]. Disrupted apical microvilli of the RPE are accompanied by photoreceptor cell death [3]. Here, we demonstrated that oxidative stress expression leads to structural changes and cellular senescence in the RPE and a decreased number of photoreceptors. However, significant improvement in the histological changes and prosenescent properties was observed with fucoxanthin treatment. The detailed mechanisms of which signaling pathways are involved in these regulations are still undergoing further studies. Besides microvilli, the actin cytoskeleton is also found at lateral contacts between epithelial cells and co-localizes with tight junctions [45]. To seal the adhesion between cells and to ensure integrity of the blood–retina barrier of tight junctions are essential to maintain visual physiology [46]. In AMD, breakdown of the blood–retina barrier, resulting in disruption of ZO-1 organization in tight junctions, increased the monolayer permeability. It is the integrity of the blood–retina barrier that keeps the choroidal vascular response from invading the retina and causing AMD [47]. We confirmed that fucoxanthin effectively stabilized the morphology of tight junctions and elevated the filamentous cytoskeleton protein F-actin in cultured RPE cells with the immunocytochemical staining assay.

The Aβs, from the amyloid β precursor protein cleaved by BACE1, are associated with the pathogenesis of Alzheimer’s disease [48]. Aβ is associated with the progression of physical disorders, and the results of several studies showed that Aβ is involved in the pathogenesis of AMD [49,50]. Studies also observed that Aβ production is involved in the development of dry AMD with the expression of some cytokines [12]. While RPE dysfunction caused by Aβ and drusen is essential for AMD pathogenesis, we determined whether fucoxanthin was involved in the oxidative stress-stimulated Aβ production. Our results show that the expression of Aβ and BACE1 decreased upon exposure to hydrogen peroxide. Since Aβ-containing elements are associated with drusen and are considered to be the initial characteristic in retinal tissues with AMD pathogenesis, decreased expression of Aβ suggests it is related to the inhibition of drusen formation. However, cross-talk between Aβ and cellular senescence was not investigated in this study. In addition, we did not examine the functional assessments of the RPE cells, including the RPE barrier or their phagocytic function. Further experiments are needed for understanding the functional assessments of RPE and photoreceptor pathogenesis.

## 4. Materials and Methods

### 4.1. Sodium Iodate-Induced Retinal Degeneration in Rat Model

Healthy 4–5-week-old male Sprague-Dawley rats (BioLASCO Taiwan, Taipei City, Taiwan) weighing 200–300 g were used. All care and treatments of experimental animal studies were approved and monitored by the Mackay Medical College Institutional Animal Care Committee (IACUC-A1070033) in accordance with institutional animal ethical guidelines. Standard diet and tap water were provided ad libitum.

The sodium iodate-induced AMD animal model has been widely used for studying retinal degeneration diseases and drug treatment effects. To induce retinal generation, experimental methods followed a previously described protocol [4] with slight modifications. Here, 50 rats were randomly split into five groups. Group I: blank control (injected phosphate-buffered saline (PBS) via the sublingual vein). Group II (injected sodium iodate (Sigma-Aldrich, St. Louis, MO, USA) via the sublingual vein at a dose of 40 mg/kg). Group III: 0.1 mg/kg fucoxanthin + sodium iodate (oral administration of 0.1 mg/kg fucoxanthin (Sigma-Aldrich) in 0.1% dimethyl sulfoxide solution mixed with 0.1 mL PBS daily for 2 weeks prior to sublingual vein injection with sodium iodate). Group IV: 1 mg/kg fucoxanthin+ sodium iodate (oral administration of 1 mg/kg fucoxanthin daily for 2 weeks prior to injection with sodium iodate). Group V: 10 mg/kg fucoxanthin+ sodium iodate (oral administration of 10 mg/kg fucoxanthin daily for 2 weeks prior to injection with sodium iodate). After 7 days of sodium iodate injection, the animals were sacrificed for follow-up experiments.

### 4.2. Staining for SA b-Gal

β-Galactosidase activity is known to be characteristic of senescent cells and is used as a biomarker for senescent and aging cells. To evaluate the protective effect of fucoxanthin on cellular senescence, galactosidase activity was analyzed. For senescence assay, experimental samples were performed according to the protocol of the SA b-Gal Staining Kit (Cell Signaling Technology, Danvers, MA). Tissue and cultured cell samples were incubated overnight at 37 °C with 5% CO_2_. The next day, β-galactosidase staining was identified by development of blue color using microscopy.

### 4.3. ROS Generation

Tissues or cells were harvested and incubated in PBS containing 10 mM general oxidative stress Indicator CM-H2DCFDA (the chloromethyl derivative of 20,70-dichlorodihydrofluorescein diacetate; Thermo Fisher Scientific, Waltham, MA, USA) for 30 min to 1 h at 37 °C in the dark to allow loading of dye into the cells. This test compound is nonfluorescent when chemically reduced, but after intracellular esterases and oxidation occur within the cell, it becomes fluorescent. The intracellular production of ROS was monitored by a microplate reader with excitation at 490 nm to obtain the absorbance value. The results were expressed as percentage of change and the blank control group was taken as 100%.

### 4.4. MDA Assay for Lipid Peroxidation

The thiobarbituric acid (TBA) reactive substances of product MDA assay (Sigma-Aldrich) was conducted for an index of lipid peroxidation and oxidative stress of retinal tissues. The method involved heating up the assay mixture comprising tissue homogenates. MDA-TBA adduct was prepared by adding TBA solution into each vial containing the standard and the sample was incubated at 95 °C for 60 min. After cooling to room temperature in an ice bath for 20 min, the reaction mixture was centrifuged for 10 min at 16,000× *g* at 4 °C. Later, the absorbance of the supernatant obtained was measured spectrophotometrically at 532 nm according to the manufacturer, and the analyzed data were expressed as malondialdehyde equivalents (nmol/mg tissue protein). These analyzed results were expressed as percentage of change and the blank control group was taken as 100%.

### 4.5. Cells and Treatments

Human RPE cell line ARPE-19 cells (American Type Culture Collection, Manassas, VA, USA) were grown in DMEM/F12 media with 10% fetal bovine serum (FBS) and standard antibiotics (100 IU/mL penicillin, and 100 μg/mL streptomycin, Sigma-Aldrich) at 5% CO_2_ and 37 °C in a humidified incubator. When cells reached confluency, cells were pretreated with various concentrations of fucoxanthin for 3 days and replaced every 24 h for the duration of the experiment. After a brief wash with medium, cells were incubated with 500 μM hydrogen peroxide in culture media for oxidative stress. All experiments were performed in triplicate.

### 4.6. MTT Assay for Mitochondrial Metabolic Rate

MTT assay (Thermo Fisher Scientific) was used to evaluate cellular metabolic activity as an indicator of cell viability and cytotoxicity. After the experimental incubation period, cells were washed once and then incubated with 0.5 mg/mL MTT labeling reagent at 37 °C for 4 h. Solubilization solution was added to solubilize the produced purple formazan crystals (MTT metabolic product). The formazan was then solubilized, and its concentration was determined by optical density at absorbance of 570 nm using a microplate reader.

### 4.7. DNA Strand Damage Marker γ-H2AX

For DNA strand damage assay, cells were fixed with 4% paraformaldehyde in PBS for 30 min at 25–27 °C and then anti-γ-H2AX (Cell Signaling Technology) at 4 °C overnight. After washing, the cells were incubated with a fluorochrome-conjugated secondary antibody at 27 °C for 1 h. After additional rinsing three times in PBS (10 min each), the cells were stained with DAPI (40,6-diamidino-2-phenylindole) nuclear probe (Roche, Basel, Switzerland) at RT for 2 min. After drying and fixation, the samples were visualized with a fluorescence microscope.

### 4.8. Scanning Electron Microscopy

Briefly, experimental cells were fixed in 2.5% paraformaldehyde and 2.5% glutaraldehyde in 0.125 M cacodylate buffer (pH 7.4) with 2 mM CaCl2. Upon postfixing with 2% osmium tetroxide in 0.1 M cacodylate buffer, experimental cells were dehydrated through a graded series of ethanol–water mixtures and then dried by the critical point method. After drying, the sample was sputter coated with gold, and cells were examined on a JEOL 100CX transmission electron microscope.

### 4.9. Immunocytochemical Staining Assay

Human RPE cell line ARPE-19 cells were fixed with 4% paraformaldehyde in PBS for 15 min, permeated in 0.05% Triton-X 100 for 15 min and blocked with 4% FBS in PBS for 30 min. Anti-ZO-1 antibody (Abcam, Cambridge, UK) was used to determine the expression of junctional proteins. Anti-Amyloid β 42 (Aβ42) (Abcam) and anti-BACE1 against β-secretase (BACE1, Abcam) antibodies were used to determine the expression of amyloid β peptides. Hoechst 33,342 or DAPI (40,6-diamidino-2-phenylindole, Thermo Fisher Scientific) was used to stain nucleic acids for the nuclear staining. Images on slides were taken using a fluorescence microscope system.

### 4.10. Statistical Analysis

Statistical data were analyzed using the SPSS program for Windows software (SPSS, Inc., Chicago, IL, USA). Means and standard deviations (SD) were presented for all experimental values in this study. The Kolmogorov–Smirnov normality test was performed to verify the normal distribution of the data. The Mann–Whitney test was used to analyze the non-parametric values. Student’s *t*-test was evaluated to compare between any two groups. One-way analysis of variance (ANOVA) followed by Dunnett’s or Bonferroni’s multiple comparison test was evaluated to analyze the parametric value groups. Statistically significant differences between groups were established when *p*-values were less than 0.05.

## Data Availability

The data presented in this study are available in the main text.
